# Compartment-specific microbial communities highlight the ecological roles of fungi in a subtropical seagrass ecosystem

**DOI:** 10.1128/aem.00606-25

**Published:** 2025-07-02

**Authors:** Xiao Wang, Jiawei Chen, Sangwook Lee, Zhicheng Ju, Anirban Akhand, Hongbin Liu

**Affiliations:** 1 Department of Ocean Science, The Hong Kong University of Science and Technology 596975, Hong Kong SAR, China; University of Delaware, Lewes, Delaware, USA

**Keywords:** fungi, prokaryotes, seagrass, microbial interdomain network, plant-microbe interaction

## Abstract

Plant-associated compartments provide habitats for various microbes. Seagrasses are the only submerged marine angiosperms. However, the simultaneous investigation of fungi and prokaryotes inhabiting different seagrass-associated compartments is limited, and much remains to be learned about the functional roles of seagrass fungi. We examined the diversity, composition, and networks of fungal and prokaryotic communities in multiple compartments associated with the seagrass *Halophila ovalis* to shed light on the significance of fungi in the seagrass ecosystem. Seagrass compartments primarily differentiated the microbial communities. Notably, the root and rhizome tissues of visually healthy *H. ovalis* exhibited a very narrow, single-species dominated and enriched fungal spectrum, leading us to hypothesize the possible establishment of a symbiotic relationship between the Lulworthiaceae mycobiont, *Halophilomyces hongkongensis*, and the seagrass host. In addition, the Vibrionaceae family, represented by the genus *Vibrio*, emerged as the most abundant prokaryotic taxon enriched in the roots/rhizomes, showing exclusive positive correlations with *H. hongkongensis* within the tissues, implying a cross-kingdom reciprocal interaction between these taxa in the endosphere of *H. ovalis*. Fungal-prokaryotic interdomain network analysis identified *H. hongkongensis* as a keystone taxon, essential for coordinating microbial interactions in *H. ovalis*-associated compartments, while robustness analysis of interdomain networks suggested fungi plays a more crucial role in sustaining the network structures of *H. ovalis* inner tissues compared to surrounding compartments. These findings provide valuable insights into the seagrass-fungi relationship and emphasize the importance of fungi in the seagrass ecosystem.

**IMPORTANCE:**

Although plant-associated microbes are key determinants of plant health, fitness, and stress resilience, microbial communities associated with seagrasses remain poorly understood compared to those in land plants, particularly concerning the diversity and ecological roles of their fungal associates. Our work provides a comprehensive assessment of fungal and prokaryotic communities across multiple above- and below-ground compartments associated with *Halophila ovalis*, the most widespread seagrass species in Hong Kong, through a year-round sampling. Our findings reveal compartment-specific patterns in diversity, network topology, and stability of microbial communities, highlighting the critical roles of fungi in seagrass-associated microbial networks and advancing our understanding of plant-fungal interactions in the marine environment.

## INTRODUCTION

Fungi and prokaryotes are major microbial groups that inhabit the plant-associated environments (1, 2). In terrestrial ecosystems, these microbes have been the particular focus of research for decades due to their critical impact on plant health, fitness, and stress resilience (3, 4). The interactions between plants and microbes have led to the consideration that plants should no longer be treated as a standalone entity but rather as a unified “holobiont” together with its microbiota (5, 6). Various factors, such as plant genotype/species, compartment, metabolism, geographic location, soil type, and environmental conditions (1, 4, 7, 8), are responsible for shaping the plant-associated microbiota. Microbe-microbe interactions also play fundamental roles in structuring the complex microbial communities in plant-associated compartments (1).

Seagrasses are the only submerged flowering plants in the marine environment. They are highly productive primary producers, often occurring in shallow seawater, and form vast meadows to provide a variety of ecological services (9, 10). Similar to terrestrial plants, seagrass tissues and surrounding compartments serve as habitats for diverse microorganisms, influencing the physiology and well-being of seagrasses. Seagrass-associated bacteria and archaea have been the target of most studies on the seagrass microbiome due to their important functions in nitrogen fixation, toxic sulfide reduction, and methane production (11–13). By contrast, our current understanding of seagrass fungi and their ecological roles is profoundly insufficient (14). Several studies have investigated microbial communities inhabiting multiple seagrass compartments (15–20), but the majority of studies only focused on a single group of microbes (either fungi or prokaryotes). Simultaneous investigation of both fungal and prokaryotic communities of seagrasses is rare, and the fungal-prokaryotic interactions in different compartments of seagrasses remain largely unknown.

Compartment differentiation of microbial diversity and composition has been frequently reported for a number of plant species (21–23), which may shed light on the potential functions or transmission modes (horizontal acquisition or vertical transmission) of certain microbial taxa in the ecosystem. For example, by comparing the microbiome of eelgrass *Zostera marina* root and leaf surfaces, together with surrounding seawater and sediments, Fahimipour et al. (24) suggested that the most abundant root-enriched genus *Sulfurimonas* (sulfur-oxidizing bacteria) may contribute to host seagrass tolerance in coastal marine habitats and possibly the recruitment of leaf bacterial epiphytes from the water column.

Understanding the ecological roles of seagrass fungi urges the exploration of their interactions with other microbial communities (14). These interactions between fungi and prokaryotes are important in shaping plant-associated microbial assemblages. They may also cause cascading consequences on plant growth and health in beneficial or deleterious ways. Such effects have been demonstrated in several land plants, including plant growth promotion, disease suppression, or facilitation (1). Network analysis based on the correlations between abundance profiles of microbial taxa enables the identification of putative cooperative or competitive associations among microbiota members and the discovery of keystone taxa that play central roles in the network. This approach has been employed in previous studies to unravel the role of fungi in microbial networks across diverse environments/samples, such as sediment (25), terrestrial plants (26, 27), and a composting system (28).

The seagrass *Halophila ovalis* (Hydrocharitaceae) has been reported to be primarily distributed across the Tropical Indo-Pacific region, as well as in southern Australia, southern Africa, and Japan (29, 30), typically characterized by paddle-shaped, small, and paired leaves. Despite its extensive global distribution, limited studies have investigated the prokaryotic communities using culture-independent methods (16, 31, 32), and information regarding its associated mycobiota is even scarcer (33). In Hong Kong, where *H. ovalis* is the prevalent seagrass species (34), local studies on its associated fungi or prokaryotes are notably lacking. Although an important initial contribution to addressing this gap has been recently provided by describing the novel fungal species and genus *Halophilomyces hongkongensis* (Lulworthiaceae) that is inextricably associated with *H. ovalis* (35), the fungal and prokaryotic communities associated with this seagrass in Hong Kong and their differentiation among multiple seagrass compartments are still poorly understood.

In this study, we used Illumina Novaseq sequencing of the internal transcribed spacer 2 (ITS2) region and the 16S rRNA gene V4 region to characterize the fungal and prokaryotic (i.e., bacterial and archaeal) communities from multiple compartments across the entire *H. ovalis* plant and surrounding environment, sampled during 4 months spanning both the dry and wet periods of a year in Hong Kong, including root, rhizome, leaf tissues, as well as phylloplane (leaf surface), rhizosphere sediment, and bulk sediment. We also examined the microbiota of seawater and non-vegetated sediment for comparisons. In addition, we investigated the fungal-prokaryotic interactions in the *H. ovalis* meadow through network analysis. From the integrated analyses of the microbial diversity and networks, we aim to describe the compartmental variation of the microbial communities associated with *H. ovalis*, to emphasize the importance of fungi in the seagrass ecosystem.

## MATERIALS AND METHODS

### Sampling and sample processing

All the *Halophila ovalis* materials used in this study were collected from the seagrass meadow located at an intertidal zone near San Tau Pier, in Tung Chung Bay, Lantau Island of Hong Kong (22°17′14″ N, 113°55′31″ E) (Fig. S1). Seagrass leaves and cores (containing below-ground seagrass tissues and adjacent sediment), together with the water and non-vegetated sediments from an area next to the seagrass meadow, were collected, using the method described by Wang et al. (35). The samples were collected from 4 months across a year, spaced to capture the two distinct periods of Hong Kong’s subtropical climate: the dry period (lower temperatures and less rainfall) and the wet period (hot, humid conditions with monsoon rains). Specifically, sampling occurred on 29 December 2022 and 10 March 2023 (dry period); 25 June and 24 August 2023 (wet period). In total, 36 seagrass cores and leaf samples, together with 20 water and 12 non-seagrass sediment samples, were obtained from the sampling. Different seagrass compartments were separated following the method reported by Wang et al. (35). After the compartment separation, 248 samples were obtained from 8 compartments, including surface-sterilized *H. ovalis* root (abbr. rt, n_rt_ = 36), rhizome (abbr. rz, n_rz_ = 36), and leaf (abbr. lf, n_lf_ = 36) tissues, leaf phylloplane (abbr. lp, n_lp_ = 36), bulk (abbr. bk, n_bk_ = 36), and rhizosphere (abbr. rp, n_rp_ = 36) sediment, as well as water (abbr. w, n_w_ = 20) and non-seagrass sediment (abbr. s, n_s_ = 12). The 248 samples were further subjected to total genomic DNA extraction for Illumina sequencing.

Seawater pH, salinity, and temperature were measured in the field during sampling using multiparameter sonde YSI EXO2s (YSI Inc., OH, USA). For sediments, the temperature was measured in the field, while pH and salinity were measured from extracted porewater using a digital pH meter DPH-2 (ATAGO, Tokyo, Japan) and an analog refractometer ORA 1SA (Kern optics, Balingen, Germany), respectively.

### DNA extraction and Illumina amplicon sequencing

For metabarcoding analyses based on Illumina sequencing, total genomic DNA from 248 samples was extracted using DNeasy PowerSoil Pro Kit (QIAGEN), DNeasy PowerBiofilm Kit (QIAGEN), and DNeasy Plant Pro Kit (QIAGEN), according to the manufacturer’s instructions. The fungal ITS2 region was amplified according to Wang et al. (35), using a nested-PCR procedure with the primer pairs ITS1F-ITS4 and fITS7-ITS4. The V4 hypervariable region of the bacterial and archaeal 16S rRNA gene was amplified using the primers 515F (5′-GTGCCAGCMGCCGCGGTAA-3′) and 806R (5′-GGACTACHVGGGTWTCTAAT-3′). PCRs were carried out in duplicate 25 µL mixture containing 2.5 µL of 10 × PCR buffer, 0.5 µL of 10 mM dNTPs, 0.75 µL of 10 mM MgCl_2_, 0.1 µL of Invitrogen Platinum Taq DNA polymerase, 0.5 µL of each 10 mM primer, and 10 ng template DNA. The PCR conditions were as follows: an initial denaturation of 95°C for 3 min, followed by 33 cycles of denaturation at 95°C for 30 s, annealing at 55°C for 30 s, extension at 72°C for 1 min, and a final extension at 72°C for 5 min. PCR products were pooled together into the library and sequenced by a NovaSeq 6000 System (Illumina Inc., San Diego, CA, USA) with 2 × 250 bp paired-end read configurations.

### Sequence processing and statistical analyses

Raw reads from Illumina NovaSeq 6000 PE250 sequencing were processed using the QIIME 2 pipeline (version 2023.2.0). The barcode- and primer-removed reads were imported to QIIME 2 (36) and demultiplexed. DADA2 (37) was used to filter low-quality reads, denoise sequences, merge read pairs, and remove chimeras. The sequences were further clustered into operational taxonomic units (OTUs) based on a 97% similarity level (38–40). The UNITE database v9.0 and SILVA database v138 were used to train naive Bayes classifiers and assign taxonomic information to the representative sequences of OTUs from the ITS2 region and the 16S rRNA gene V4 region Illumina sequencing, respectively. The raw sequencing fastq files were deposited in the Sequence Read Archive (SRA) at the National Center for Biotechnology Information (NCBI) as BioProject ID PRJNA1062533.

All statistical analyses were performed using R version 4.3.2. The output OTU count tables, taxonomy tables, and phylogenetic trees were compiled in Phyloseq R files (41). The OTU tables were rarefied to the minimum sequencing depth for downstream analyses. Rarefaction curves were drawn to reveal the sufficiency of sequencing depth. Permutational multivariate analysis of variance (PERMANOVA) was used to analyze the relative importance of categorical (compartment and sampling month) and environmental (pH, salinity, and temperature) factors on the fungal and prokaryotic communities (42, 43). Alpha-diversity indices (observed OTU numbers and Shannon index) were calculated to estimate the richness and diversity of microbial communities from different compartments and compared by Wilcoxon rank-sum tests. Relative abundance of fungi at the phylum and genus levels, as well as prokaryotes at the phylum and family levels, was calculated and visualized by bar plots using microViz (v 0.11.0) to show the abundant taxa in multiple micro-habitats. The differences between fungal and prokaryotic phyla among compartments were evaluated by the Kruskal-Wallis test. Principal coordinates analysis (PCoA) and analysis of similarities (ANOSIM) with 999 permutations were performed for the assessment of dissimilarities among microbial communities in below- and above-ground compartments based on Bray-Curtis distance.

To find out the significantly enriched taxa in *H. ovalis* meadow, we performed SIMPER analysis in the “vegan” package (version 2.6-4) to compare the changes of relative abundance for fungi and prokaryotes between compartments. The comparisons included the following pairs: non-seagrass sediment versus seagrass sediment, seagrass sediment versus below-ground tissues, also water versus leaf surface, and water versus leaf tissue. During the calculation of fold change, the 0 values in the denominator were changed to the lowest non-zero relative abundance in that compartment to avoid an undefined fraction. Only the taxa with relative abundance higher than 0.01%, representing non-rare taxa in the latter compartment (numerator) of each pair, were presented in the plots.

### Network construction and analysis

Fungal-prokaryotic whole (including all types of connections between nodes) and interdomain (only including connections between fungi and bacteria/archaea) networks were constructed and analyzed, respectively, from *H. ovalis* leaf, root, rhizome tissues, as well as leaf phylloplane and seagrass sediment, to assess network variability among compartments. The networks were constructed using the integrated Network Analysis Pipeline (iNAP) (44) based on the SparCC method. Only OTUs present in more than 50% of replicates were included for correlation calculation. A statistical significance threshold of 0.05 was set, and non-correlated edges were removed under the threshold value of 0.2 to generate the networks. The topological properties of the networks, such as the nodes, edges, node degree, and modularity, were calculated to characterize the structure of the networks. All networks were visualized using Gephi 0.10. The keystone taxa were identified by the *Z_i_-P_i_* (within- and among-module connectivity) plots based on the contribution of nodes within each interdomain network and categorized into Connectors, Module hubs, and Network hubs.

To characterize the network stability, the robustness of networks was calculated and compared. Robustness is defined as the proportion of nodes remaining in the networks after the random removal of nodes, based on the calculation of the abundance-weighted mean interaction strength of nodes (45). Here, we only focused on the scenario of fungal node loss to understand the contribution of fungi to the stability of the constructed interdomain networks.

## RESULTS

### Processing of amplicon sequencing data

For fungal ITS2 region Illumina sequencing, 51,135,714 raw reads and 38,263,577 effective sequences were generated. The number of sequences from the samples ranged from 43,989 to 466,926. After the removal of non-fungal sequences (unassigned and other non-fungal eukaryotic sequences) and low-read samples, 247 samples remained and were rarefied to 2,291 sequences per sample with 4,322 OTUs. A total of 40,159,902 raw reads and 26,433,563 effective sequences were generated from the 16S rRNA gene V4 region Illumina sequencing using the NovaSeq 6000 PE250 platform. The number of sequences from the samples ranged from 56,417 to 176,224. After the removal of non-prokaryotic sequences (chloroplast, mitochondria, unassigned) and low read samples, 245 samples remained and were rarefied to 5,488 sequences per sample with 20,577 OTUs classified into bacteria (18,988 OTUs) and archaea (1,589 OTUs). The fungal and prokaryotic OTU count and taxonomic information are reported at https://github.com/wangxiaobiol/seagrassmicrobiome. Rarefaction curves of OTUs (Fig. S2) showed all the curves approaching saturation, indicating a sufficient sequencing depth.

### Compartment-specific diversity of microbial communities

The measured physicochemical parameters, including pH, temperature, and salinity (Fig. S3), along with categorical factors (compartment and sampling month), had different levels of influence on fungal and prokaryotic communities. Permutational multivariate analysis (Table S1) revealed that the structure of both fungal and prokaryotic communities was mainly explained by compartment, which had a greater impact on the investigated prokaryotic community (*r*^2^ = 0.4868, *P* = 0.001) than on the fungal community (*r*^2^ = 0.2419, *P* = 0.001). While sampling month, pH, temperature, and salinity also significantly influenced microbial community structure, with less pronounced effects than those of the compartment.

**Fig 1:**
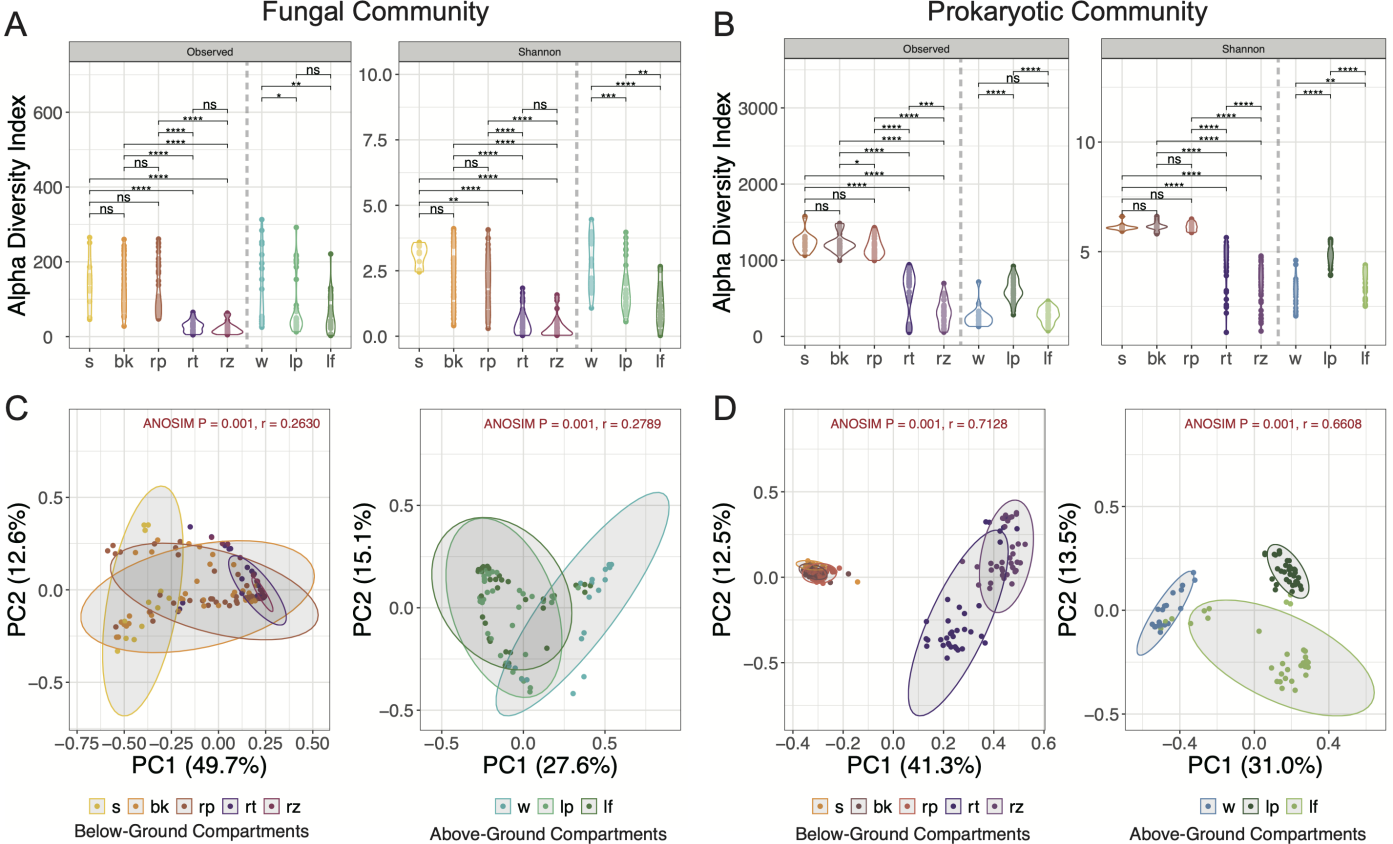
Comparisons of alpha- and beta-diversity of fungal and prokaryotic communities from multiple compartments. (**A, B**) Violin plots show the alpha-diversity indices (Shannon index and observed OTU numbers) of fungal and prokaryotic communities, respectively. The left side of the dashed line includes below-ground compartments, while the right side includes above-ground compartments. Differences between compartments evaluated by Wilcoxon rank-sum test indicated as: *P < 0.05, **P < 0.01, ***P < 0.001, ****P < 0.0001, ns = not significant. (**C, D**) Principal coordinates analysis (PCoA) plots of fungal and prokaryotic communities based on Bray–Curtis distance, respectively. The *r* and *P*-values of analysis of similarities (ANOSIM) were shown in each figure. For different compartments, s = non-seagrass sediment, bk = bulk sediment, rp = rhizosphere sediment, rt = root, rz = rhizome, w = water, lp = leaf phylloplane, lf = leaf.

Alpha and beta diversity of fungal and prokaryotic communities were analyzed. For below-ground compartments, all the sediments (*H. ovalis* bulk and rhizosphere sediment, as well as non-seagrass sediment) had more diverse and richer microbial communities than *H. ovalis* roots and rhizomes, supported by *P*-values less than 0.0001 (Fig. 1A and B). Among above-ground compartments (water, *H. ovalis* leaf tissue, and phylloplane), the alpha diversity of fungal communities decreased significantly from water to leaf tissue and phylloplane (Fig. 1A), while the highest alpha diversity of prokaryotic communities was found from phylloplane (Fig. 1B). Both fungal and prokaryotic communities inhabiting the below- and above-ground compartments were clearly distinguished according to principal coordinates analysis (PCoA) based on Bray-Curtis distance (ANOSIM *P* = 0.001) (Fig. 1C and D).

### Distinct taxonomic composition of microbial communities among compartments

**Fig 2:**
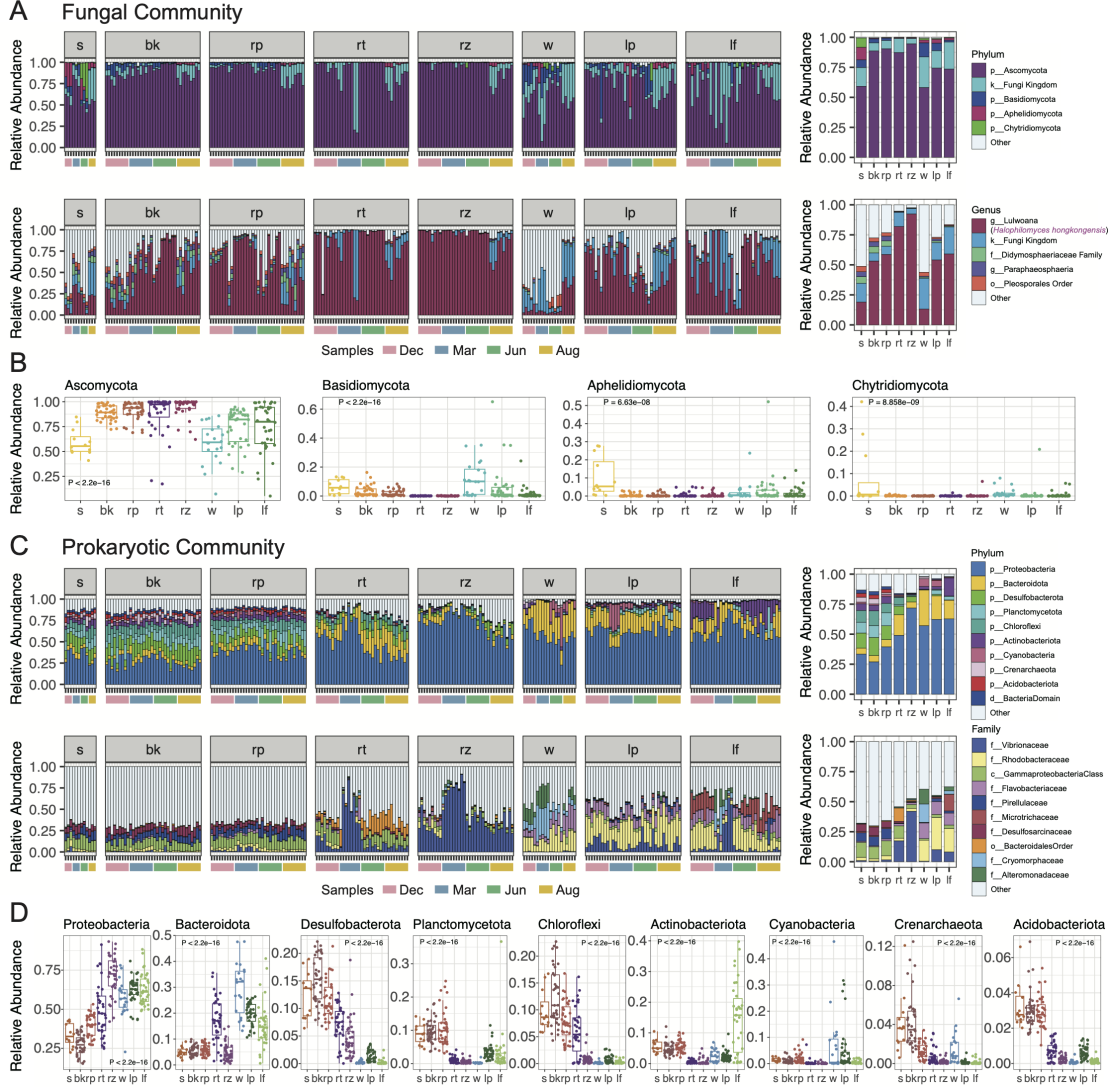
Taxonomic composition of fungal and prokaryotic communities. (**A**) Relative abundance of fungi at phylum and genus levels. (**B**) Comparisons of the most abundant fungal phyla in different compartments by the Kruskal-Wallis test. (**C**) Relative abundance of prokaryotes at phylum and family levels. (**D**) Comparisons of the most abundant prokaryotic phyla in different compartments by the Kruskal-Wallis test. The top five fungal phyla and genera, as well as the top ten prokaryotic phyla and families, are presented; other taxa with lower abundances were merged as “other.” In the taxa names, “d” = domain, “k” = kingdom, “p” = phylum, “c” = class, “o” = order, “f” = family, “g” = genus. In the compartment names, s = non-seagrass sediment, bk = bulk sediment, rp = rhizosphere sediment, rt = root, rz = rhizome, w = water, lp = leaf phylloplane, lf = leaf. In this figure, the original fungal genus “*Lulwoana*” classified based on UNITE Database was replaced by the recently described novel taxon *Halophilomyces hongkongensis* according to Wang et al. (35), because the “*Lulwoana*” representative OTU clustered into an independent clade with *H. hongkongensis* isolates.

The relative abundance of fungal communities at phylum and genus levels in multiple compartments is shown in Fig. 2A. The most abundant fungal phyla, including Ascomycota, Basidiomycota, Aphelidiomycota, and Chytridiomycota, presented significant compartment-specificity (Kruskal-Wallis test *P* < 0.01) (Fig. 2B). Compared to non-seagrass sediment and seawater, Basidiomycota occupied relatively smaller proportions in seagrass-associated compartments. By contrast, Ascomycota exhibited a different distribution pattern, with lower proportions observed in non-seagrass sediment and water (Fig. 2A and B). Furthermore, non-seagrass sediment fungal communities were featured by the highest relative abundances of Aphelidiomycota and Chytridiomycota compared to other compartments (Fig. 2A and B). The genus “*Lulwoana*,*”* which actually corresponds to the recently described novel taxon *H. hongkongensis* (35), was found in extremely high proportions within the rhizomes (92.20%) and roots (81.96%) of *H. ovalis*, with lower proportions found in seagrass surrounding sediments, leaf tissue, and phylloplane (53.16%–59.06%). Non-seagrass sediment and water had the least abundance of *H. hongkongensis* (below 19.03%) (Fig. 2A). In this study, the originally classified genus “*Lulwoana”* has been changed to *H. hongkongensis* based on phylogenetic evidence according to Wang et al. (35), as the representative OTU of “*Lulwoana”* formed a distinct clade with *H. hongkongensis* isolates. In addition, the genus *Paraphaeosphaeria*, along with other taxa identified only at the kingdom (Fungi), family (Didymosphaeriaceae), or order (Pleosporales) levels, was also abundant (Fig. 2A).

Prokaryotic composition at the phylum level also significantly differed among compartments (Kruskal-Wallis test; *P* < 0.0001) (Fig. 2C and D). Proteobacteria was the most dominant prokaryotic phylum across seagrass and non-seagrass area (Fig. 2C). At the family level, Vibrionaceae dominated the *H. ovalis* rhizomes (42.06%) and roots (17.44%), followed by phylloplane (10.07%) and leaf tissues (8.18%), with much lower proportions observed in all types of sediments and water (less than 1.62%) (Fig. 2C). The family Vibrionaceae was represented by the genus *Vibrio*, which comprised 98.21% of the total Vibrionaceae abundance (Table S2). Two main *Vibrio* OTUs contributed significantly to this abundance, accounting for 50.14% and 35.54% of the *Vibrio* community, respectively, followed by two other OTUs occupying 8.13% and 6.11% (Table 1; Table S3). A higher percentage of Desulfosarcinaceae was discovered in sediments than in other compartments. Rhodobacteraceae and Flavobacteriaceae represented significant portions of the above-ground prokaryotic community. Microtrichaceae occupied important proportions in leaf internal tissues (Fig. 2C).

**TABLE 1:** Relative abundance of OTUs belonging to the genus *Vibrio*

*Vibrio* species	OTU ID	Proportion in the *Vibrio* community
*Vibrio* sp.1	POTU11812	50.14%
*Vibrio* sp.2	POTU11821	35.54%
*Vibrio* sp.3	POTU11814	8.13%
*Vibrio* sp.4	POTU11815	6.11%
Other *Vibrio* spp.	Other 29 OTUs	0.08%

### Enrichment of microbial taxa in *H. ovalis*-associated compartments

**Fig 3:**
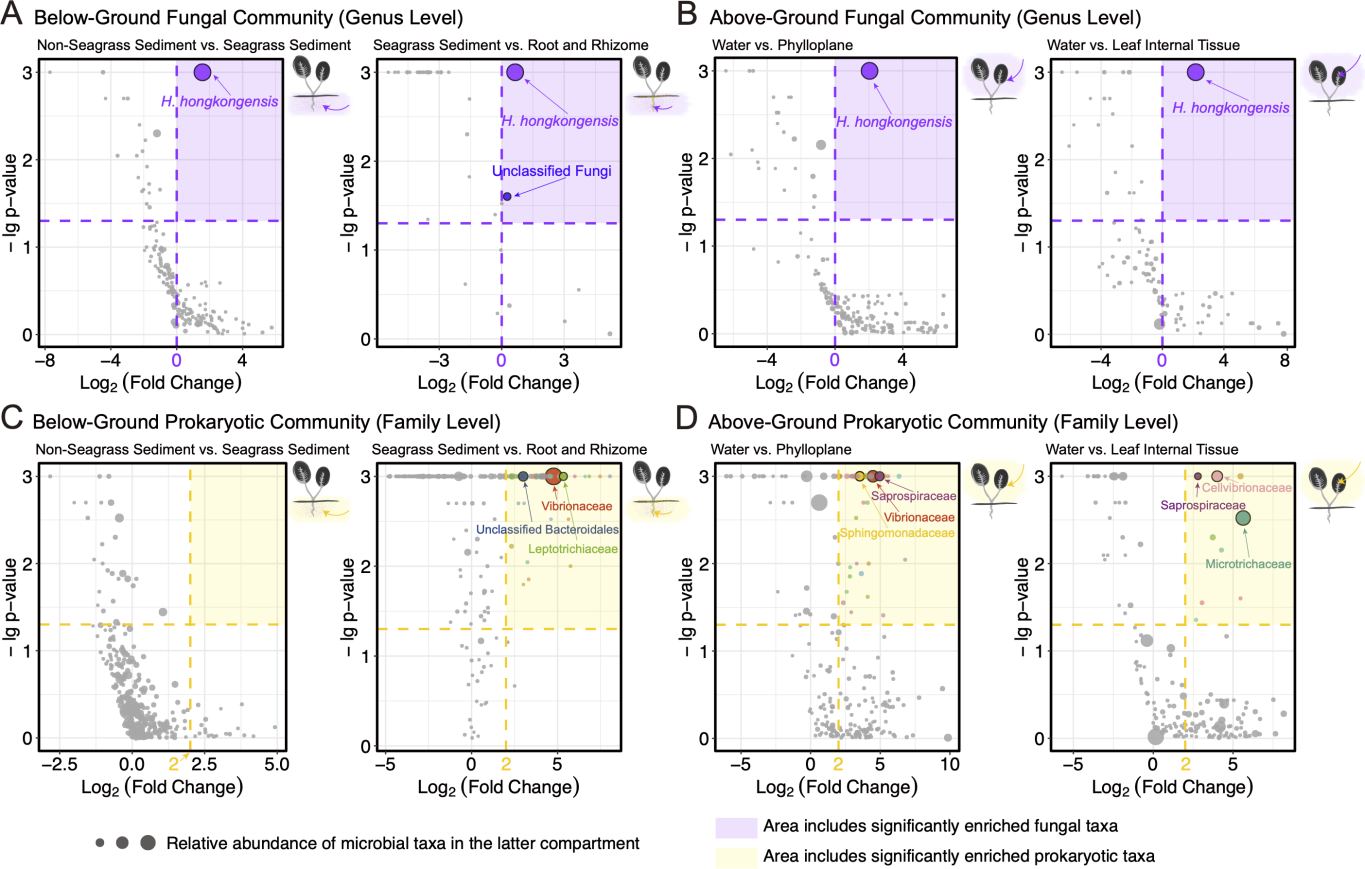
Enriched fungal (**A, B**) and prokaryotic (**C, D**) taxa in seagrass *Halophila ovalis* surrounding compartments and inner tissues, by comparing the fold changes of microbial taxa relative abundance from non-seagrass sediment to seagrass sediment, from seagrass sediment to root and rhizome, and from water to phylloplane, also from water to leaf tissue. Fold changes and *P*-values were calculated using SIMPER analysis. The cut-off for *P*-value is 0.05. For the fungal community, the cut-off for Log_2_(Fold Change) is 0, while it is 2 for the prokaryotic community. Each dot represents a specific fungal or prokaryotic taxon. The size of the dot corresponds to its relative abundance in the latter compartment from each pair of comparisons. The purple and yellow shaded areas in subplots include significantly enriched fungal and prokaryotic taxa, respectively. The significantly enriched taxa with the highest relative abundances are marked with their taxonomic identification in the subplots.

To further reveal the compartment-specific composition of the microbiome and investigate the fungi and prokaryotes that share close relationships with the seagrass *H. ovalis*, we compared the changes in relative abundance for all fungal and prokaryotic taxa between different below/above-ground compartments (Fig. 3).

As for the fungal community, overall, *H. hongkongensis* was the sole fungal taxon enriched in *H. ovalis* tissues and surrounding compartments (Fig. 3A and B). It exhibited a continuously increasing trend from non-seagrass sediment to seagrass surrounding sediment (Fold Change = 2.9387, *P* = 0.001) to below-ground roots and rhizomes (Fold Change = 1.5597, *P* = 0.001) (Fig. 3A; Table S4). Similarly, for above-ground compartments, *H. hongkongensis* was also remarkably enriched in leaf surface (Fold Change = 4.0956, *P* = 0.001) and tissue (Fold Change = 4.4721, *P* = 0.001) compared with the surrounding water (Fig. 3B; Table S4). The relative abundance of unclassified fungi that were only identified to the kingdom level was also significantly increased from seagrass sediment to below-ground tissues (Fold Change = 1.1975, *P* = 0.025) (Fig. 3A; Table S4), which did not provide much information because it may be composed of multiple fungal genera.

There was no significant increase in the relative abundance of any prokaryotic family from non-seagrass to seagrass sediment (Fig. 3C). Fifty-seven prokaryotic families were apparently enriched in *H. ovalis* below-ground tissues versus surrounding sediments, of which Vibrionaceae (29.92%), unclassified Bacteroidales (6.00%), and Leptotrichiaceae (3.78%) are the most abundant (Fig. 3C; Table S5). For above-ground compartments, among the 35 significantly increased prokaryotic families on the leaf surface compared to water, Vibrionaceae (10.07%), Sphingomonadaceae (5.21%), and Saprospiraceae (4.72%) occupied the largest proportions (Fig. 3D; Table S5). The leaf internal tissue was featured by the enrichment of Microtrichaceae (13.87%), Cellvibrionaceae (6.27%), and Saprospiraceae (1.06%), together with six other families (Fig. 3D; Table S5).

### Fungal-prokaryotic network structure and stability vary among compartments

The fungal-prokaryotic whole networks from the five *H. ovalis*-associated compartments showed dramatic topological differences (i.e., nodes, edges, average degree, and modularity). The whole networks of *H. ovalis* phylloplane and sediment were represented by significantly higher values of nodes, edges, average degree (*P* < 0.0001), and lower modularity than the inner tissues (Fig. S4A through F; Table 2). Fungi and prokaryotes themselves are more intended to correlate positively, as revealed by the generally higher number of positive edges in the fungal and prokaryotic networks of different compartments, respectively, whereas more negative edges were found between fungi and prokaryotes (Fig. S4G).

**TABLE 2:** Topological properties of fungal-prokaryotic whole and interdomain networks across *Halophila ovalis*-associated compartments^*a*^

Networks	Total nodes	Total edges	Positive edges	Negative edges	Modularity	Average degree
Whole networks						
Leaf	163	2,970	1,504	1,466	0.263	36.442
Root	255	4,773	2,392	2,381	0.264	37.435
Rhizome	139	2,330	1,155	1,175	0.246	33.525
Phylloplane	350	14,633	7,267	7,366	0.221	83.617
Seagrass sediment	742	27,583	14,293	13,290	0.204	74.348
Interdomain networks						
Leaf	139	363	158	205	0.362	5.223
Root	133	174	70	104	0.432	2.617
Rhizome	86	105	53	52	0.434	2.442
Phylloplane	305	1,312	610	702	0.280	8.603
Seagrass sediment	536	2,470	1,012	1,458	0.345	9.216

^*a*^ The networks were constructed based on the SparCC method (|*r*| > 0.2, *P* < 0.05). Only OTUs present in more than 50% of samples were included for correlation calculation.

**Fig 4:**
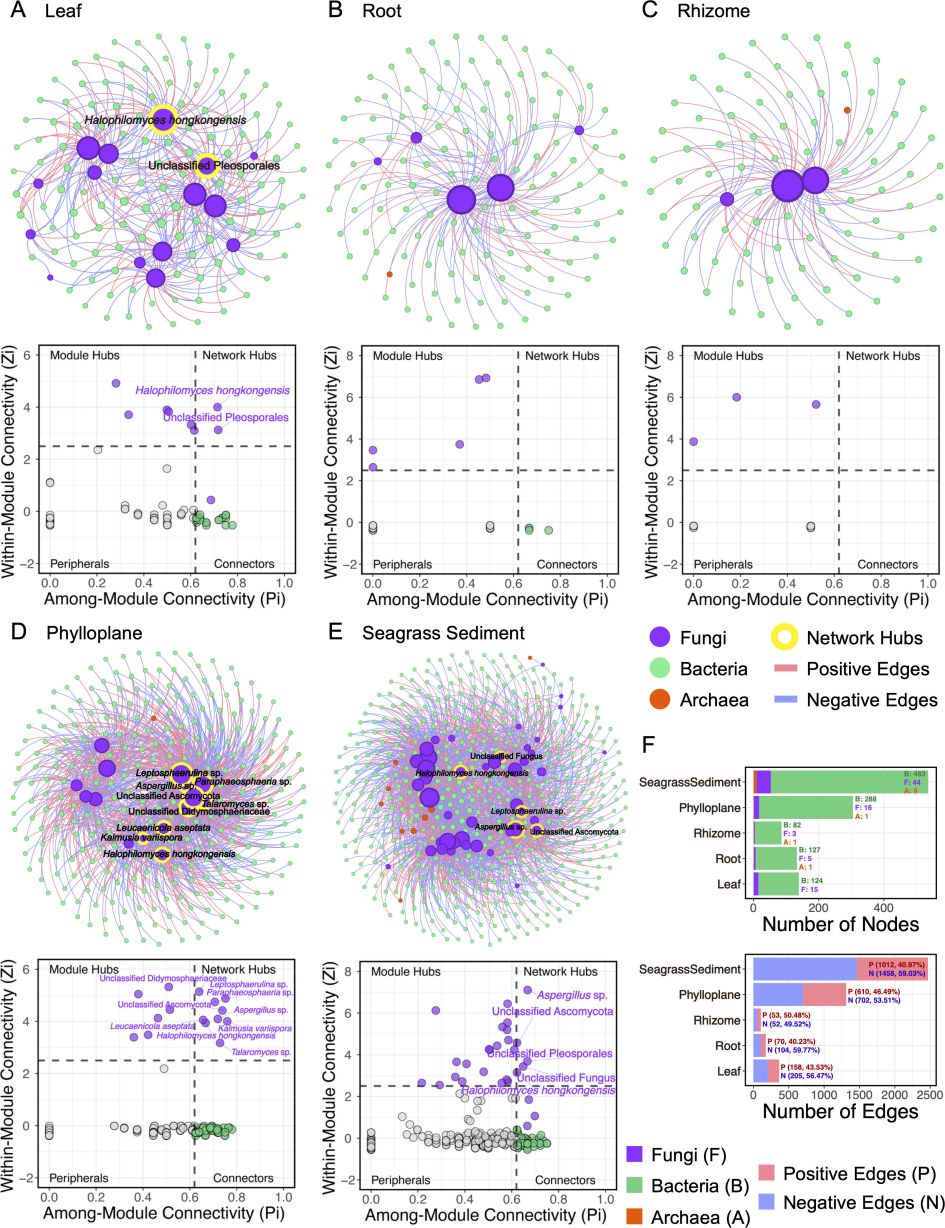
The structure of fungal-prokaryotic interdomain networks and *Z_i_-P_i_* plots of OTUs in the seagrass *H. ovalis* leaf (**A**), root (**B**), rhizome (**C**), phylloplane (**D**), and sediment (**E**). Interdomain networks only include edges between fungi and prokaryotes. The node size in interdomain networks is proportional to the degree (number of direct correlations to a node). 2.5 and 0.62 were set as the threshold values for *Z_i_* and *P_i_* to categorize keystone OTUs, respectively. Only the Network hubs are marked with their taxonomic identification in the subplots. (**F**) The number/proportion of fungal, bacterial, and archaeal nodes, as well as the positive and negative edges in each interdomain network.

**Fig 5:**
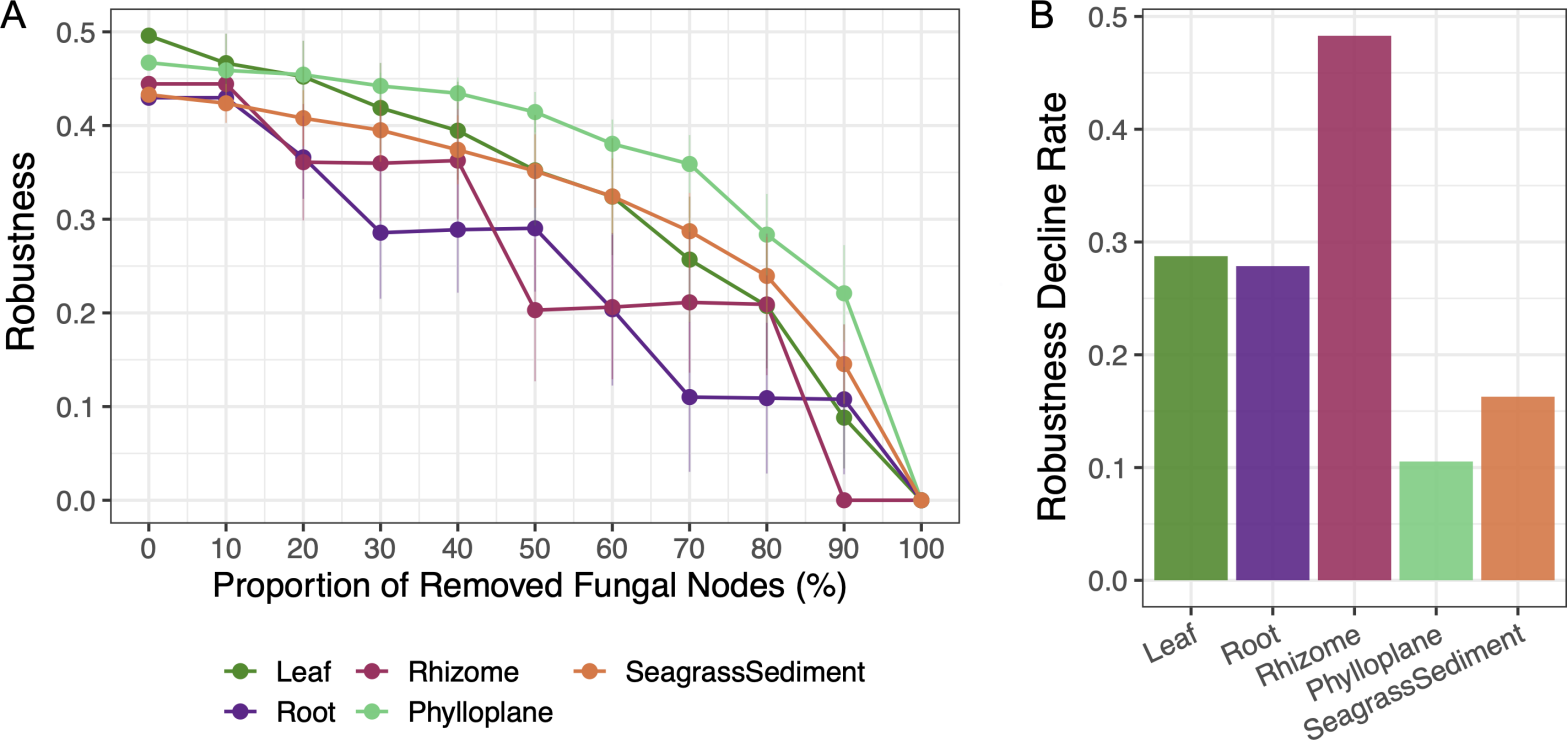
The resistance of interdomain networks to fungal node loss. (**A**) Robustness when randomly removing fungal nodes. Robustness is defined as the proportion of nodes remaining in the interdomain networks after the random removal of certain nodes. Each dot represents the mean robustness for 1,000 randomly generated networks, and bars correspond to the standard deviation. (**B**) The robustness decline rate of each interdomain network, measured as the rate of robustness change after 50% of nodes were randomly removed, equals the absolute value of ΔRobustness/0.5.

Elucidation of the interactions between fungi and other organisms is crucial for understanding the poorly defined role of fungi in certain ecosystems (25, 28). The fungal-prokaryotic interdomain networks showed compartment-specific topology similar to those of the whole networks, with the outer compartments of *H. ovalis* involving more nodes and edges, also exhibiting higher average degree and lower modularity (Fig. 4; Table 2). Keystone nodes that contribute significantly to coordinating the network structure were discovered as presented by the *Z_i_-P_i_* plots (Fig. 4). The number of keystone taxa in the five interdomain networks was 36 (leaf), 9 (root), 3 (rhizome), 112 (phylloplane), and 104 (sediment) (Fig. 4; Table S6). Most keystone fungi served as either Module hubs (in all compartments) or Network hubs (in leaf, phylloplane, and sediment), some as Connectors (only in leaf and sediment), while keystone prokaryotes were all acting as Connectors (Fig. 4; Table S6). We noticed the predominant fungus *H. hongkongensis* acted as the keystone taxon in all compartments, serving as network hubs in the *H. ovalis* leaf, phylloplane, and sediment while functioning as module hubs inside the roots and rhizomes (Fig. 4; Table S6). The stability of interdomain networks varied across compartments. During the random removal of fungal nodes from the interdomain networks, the robustness of all networks decreased (Fig. 5A) but at different rates (Fig. 5B). The removal of fungi caused more pronounced effects on the robustness of *H. ovalis* inner tissues compared to the surrounding compartments, as reflected by the more rapid decline in robustness curves for the inner tissues (decline rate: rhizome >leaf > root) than for the phylloplane and sediment (decline rate: sediment >phylloplane) (Fig. 5), illustrating more stable interdomain network structures for phylloplane and sediment than the inner tissues.

### Positive correlations between *H. hongkongensis* and *Vibrio* spp. inside *H. ovalis*

**Fig 6:**
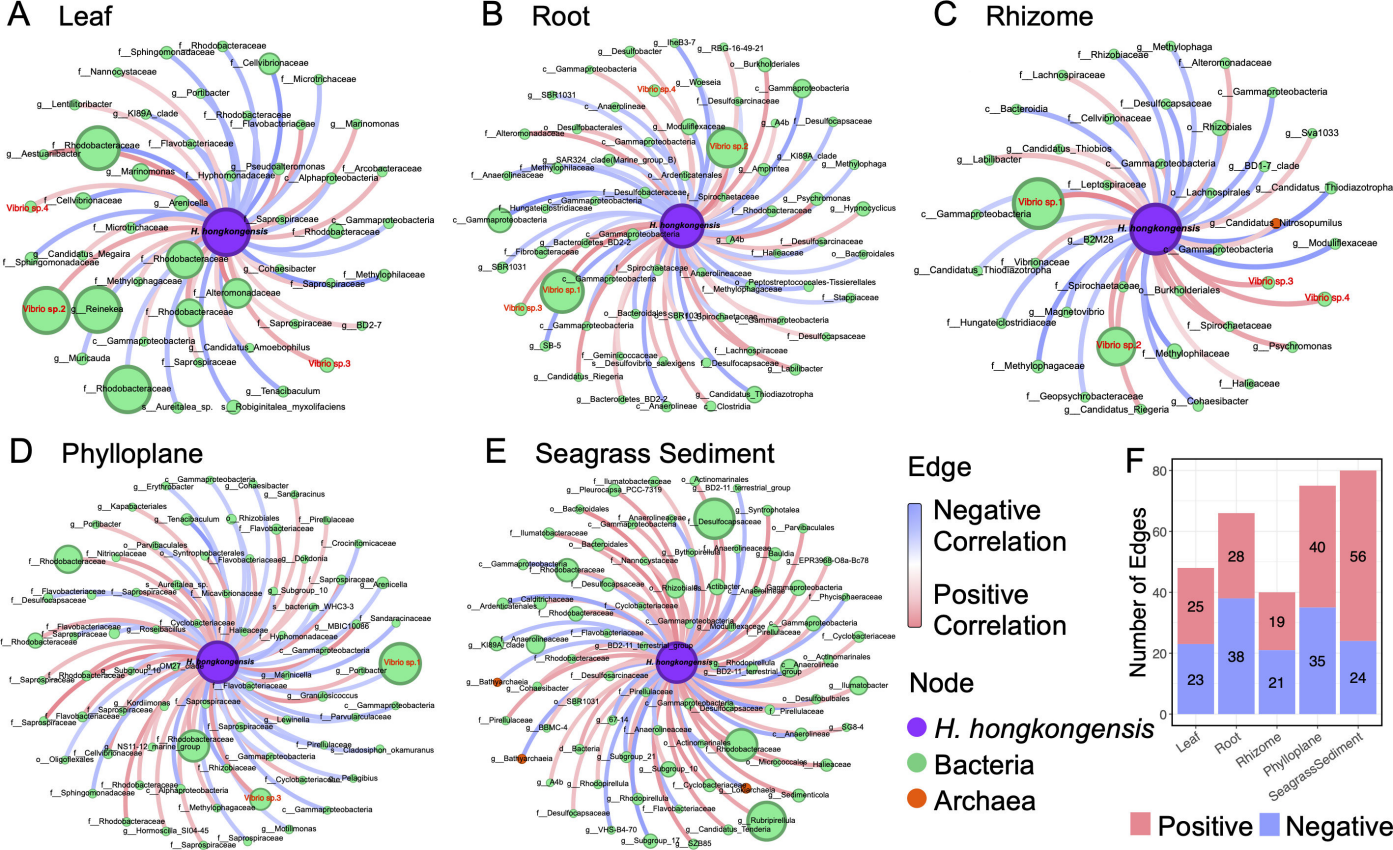
Networks of prokaryotic OTUs significantly correlated with the key fungus *Halophilomyces hongkongensis* in the seagrass leaf (**A**), root (**B**), rhizome (**C**), phylloplane (**D**), and sediment (**E**). The size of a certain prokaryotic node corresponds to its relative abundance, and the size of *H. hongkongensis* is fixed. (**F**) Number of positive and negative correlations between prokaryotes and *H. hongkongensis* in each seagrass compartment. In the prokaryotic taxa names, “d” =domain, “c” =class, “o” =order, “f” =family, “g” =genus, “s” =species.

To further determine the potential roles of the key fungus *H. hongkongensis* in the investigated *H. ovalis* meadow, we zoomed in on the networks that only include correlations between *H. hongkongensis* and prokaryotes (Fig. 6). *H. hongkongensis* correlated with 48 (leaf), 66 (root), 40 (rhizome), 75 (phylloplane), and 80 (sediment) prokaryotes in different compartments, respectively, with a higher number of negative edges than positive ones detected from *H. ovalis* roots (38 vs. 28) and rhizomes (21 vs. 19) (Fig. 6F). We found the genus *Vibrio,* which predominated the prokaryotic community of *H. ovalis* below-ground tissues (Fig. 2C), only positively interacted with the key fungus *H. hongkongensis* inside the seagrass (Fig. 6A through C). For the below-ground tissues (root and rhizome), *H. hongkongensis* had significantly positive correlations with the four major *Vibrio* species *Vibrio* sp.1, *Vibrio* sp.2, *Vibrio* sp.3, and *Vibrio* sp.4 (correlation value 0.91, 0.69, 0.73, and 0.72 for rhizome, 0.64, 0.68, 0.51, and 0.33 for root, respectively) (Fig. 6B and C; Table S7). Similarly, *H. hongkongensis* inhabiting the leaf also exclusively established positive correlations with *Vibrio* spp., including *Vibrio* sp.2 (correlation value 0.47), *Vibrio* sp.3 (correlation value 0.45), and *Vibrio* sp.4 (correlation value 0.33) (Fig. 6A; Table S7). By contrast, a negative correlation was found between *Vibrio* sp.1 and *H. hongkongensis* on the leaf surface (Fig. 6D; Table S7). In addition, we noticed several archaeal taxa significantly correlated with *H. hongkongensis*, including *Candidatus Nitrosopumilus* (inside rhizome, positive correlation) (Fig. 6C; Table S7), as well as *Lokiarchaeia* and *Bathyarchaeia* (in seagrass sediment, negative correlations) (Fig. 6E; Table S7).

## DISCUSSION

This study provides an unprecedented investigation of both fungal and prokaryotic communities and their interdomain associations in multiple seagrass-associated compartments. We observed compartment-specific fungal and prokaryotic communities associated with the seagrass *H. ovalis* in terms of taxonomic composition, network topology, and stability. Plant-associated compartment is regarded as a proxy of variables that differ in resource availability, physicochemical properties, and structural characteristics (43). Specifically, we found that the compartment had a more profound effect in shaping the structure of prokaryotic communities (PERMANOVA *r*^2^ = 0.4868) compared to fungal communities (PERMANOVA *r*^2^ = 0.2419). This result aligns with previous studies on terrestrial plants, which similarly reported the larger role that plant compartments played in shaping the prokaryotic communities compared to the fungal communities (7, 46).

The significantly more diverse and richer fungal/prokaryotic communities in surrounding compartments (sediments and phylloplane/water) than the inner tissues (leaf, root, and rhizome) of *H. ovalis* are consistent with the generally known distributional pattern of microbiota in plant holobiont (21, 47, 48), indicating a filter mechanism of seagrass that only selects specific microbial taxa (4, 21, 46, 49). Prokaryotes exhibited higher alpha diversity on the leaf surface compared to the water, while fungal communities showed a contrasting pattern, suggesting that the leaf surface of *H. ovalis* serves as a hotspot for the colonization of diverse prokaryotes. In general, the leaf surface of seagrass is mostly colonized by heterotrophic prokaryotes that are usually involved in polymer degradation, surface attachment, and biofilm formation (11). Indeed, we noticed that such prokaryotic taxa enriched and dominated the phylloplane of *H. ovalis*. For instance, some members of Sphingomonadaceae were previously reported to be able to degrade xenobiotic and recalcitrant (poly)aromatic compounds (50, 51), or being the dominant components of biofilms (52–54); Saprospiraceae is known to break down complex organic carbon (55, 56); and the genus *Vibrio* (representing the family Vibrionaceae) has demonstrated versatile metabolic capabilities (57–59). The fungal-prokaryotic networks also showed compartment specificity. We observed more complex fungal-prokaryotic networks in *H. ovalis* sediment and phylloplane than in tissues, as indicated by the number of nodes, edges, and the average degree. The nutrient exchange and plant metabolites surrounding the seagrass could attract abundant microbes and enable these habitats to be dynamic interfaces (21, 60–62), thereby promoting microbes to cooperate or compete for food and resources. Besides, the higher modularity of the leaf, root, and rhizome networks compared to the surrounding compartments may be attributed to the more heterogeneous and complex habitat architecture (i.e., compartment internal structure) in tissues than the phylloplane and sediment (62, 63).

The root/rhizome tissues of *H. ovalis* were stably colonized by a narrow range of fungi, predominantly by a single species, *H. hongkongensis*, which belongs to the obligate marine Lulworthiaceae family and exceeded 80% in relative abundance. Further SIMPER analyses revealed that *H. hongkongensis* was the sole fungal taxon enriched within the *H. ovalis* tissues, emphasizing its uniqueness and importance to the host. The extreme dominance by a single fungal species is unusual for below-ground tissues of most photoautotrophic plants, where endophytic fungal communities typically exhibit higher diversity, as demonstrated in mangroves (64), freshwater aquatic plants (65, 66), herbs and shrubs (62, 67), and even other seagrasses (e.g., *H. ovalis* [33] and *Enhalus acoroides* [68] from Singapore and peninsular Malaysia). A similar example of a single-species-dominated seagrass root mycobiome can be found in the Mediterranean seagrass *Posidonia oceanica*, where the dark septate endophytic fungus *Posidoniomyces atricolor*, representing a marine lineage in the Aigialaceae family (Pleosporales), is the major root mycobiont (69, 70). In our study, the distribution of *H. hongkongensis* was not confined to the root region but also extended to the rhizomes and leaves; in addition, the *H. hongkongensis* strains were obtained from common media and culture conditions for fungal isolation (35). These observations suggest that the relationship between *H. ovalis* and its fungal partner may not be highly specialized, as strict symbiotic/mutualistic partners (e.g., mycorrhizal fungi) are typically root-restricted and demand specific conditions for isolation (71–73). Although there is currently no solid proof that *H. hongkongensis* inside *H. ovalis* is beneficial to the host plant, its regular dominance and unique enrichment in visually healthy seagrass specimens suggest a possible general symbiotic relationship between this marine Lulworthiaceae member and its seagrass host.

Previous studies have demonstrated the ecological and biotechnological potential of Lulworthiaceae fungi through different aspects. The members of Lulworthiaceae have been hypothesized to have a lignicolous nature, supported by their association with specific substrates (typically seagrasses [69, 74, 75], seaweeds, and submerged/drifted woods [74, 76, 77]) and their ability to produce lignocellulolytic enzymes (78). These traits may indicate their ecological role in marine nutrient cycling through the degradation of plant biomass (wood components and marsh plants), with potential extension to anthropogenic materials, such as microplastics and polycyclic aromatic hydrocarbons (74, 75, 79). In addition, some Lulworthiaceae members exhibit antibacterial properties, including activity against methicillin-resistant *Staphylococcus aureus* (35, 77), with the recent isolation of the antibacterial compound lulworthinone (77) confirming their potential as a source of marine natural drug candidates.

Plant-associated microbial networks tend to indicate the dominance of negative correlations between microbes belonging to different kingdoms, whereas positive correlations primarily occur through intra-kingdom networks (80). In line with this pattern, our analyses revealed that negative correlations between fungi and prokaryotes were more commonly established, while positive correlations are likely to prevail among either fungal or prokaryotic microbes. The aforementioned correlation pattern suggests competitive mechanisms might be primarily favored by microbial groups from different kingdoms that are phylogenetically distant, as opposed to closely related taxa (1). During the random removal of fungal nodes from *H. ovalis*-associated fungal-prokaryotic interdomain networks, we noticed a more rapid decline of robustness in *H. ovalis* tissues, indicating fungi played a more crucial role in maintaining the interdomain network structures of *H. ovalis* inner tissues than surrounding compartments. Plant-associated microbial interdomain networks generally contain fewer fungi than prokaryotes (26, 81, 82). Although the interdomain networks within the inner tissues of *H. ovalis* contain only a small number of fungi, these fungi are regular inhabitants of the seagrass endosphere. The deletion of these fungal nodes can lead to devastating consequences for the fungal-prokaryotic interactions in these micro-habitats, ultimately affecting the entire microbial network that is important to plant health and ecosystem functioning (83).

Network analysis enabled us to identify the keystone species, *H. hongkongensis*, which acted as a pivotal component for coordinating the structure of the fungal-prokaryotic interdomain networks. Given its importance as a keystone species, we subsequently focused on the prokaryotes correlated with this species. We found it particularly noteworthy that the key fungus established exclusively positive links with species from the most abundant root/rhizome- and phylloplane-enriched prokaryotic genus, *Vibrio,* inside different vegetative organs of *H. ovalis*, in contrast to the negative link observed on the phylloplane of *H. ovalis*. The beneficial effects of endophytic *Vibrio* species toward seagrass hosts have been previously demonstrated, including nitrogen fixation or being involved in the production of plant hormone IAA (indole-3-acetic acid) (84, 85). The compartment-specific inter-microbial interaction we discovered suggests a cooperative strategy between *H. hongkongensis* and *Vibrio* spp. in the endosphere of *H. ovalis*, which may further influence the host growth and fitness. The differences in micro-environmental conditions might cause the compartment-specific *H. hongkongensis-Vibrio* relationship, as the seagrass endosphere is a more sheltered, stable, nutrient-rich, and less exposed environment compared to the surrounding environments. This distinct environment may result in different interaction dynamics and favor the positive correlations between *H. hongkongensis* and *Vibrio* species. The cooperation between fungi and bacteria in land plants is common; for example, root mycorrhizal fungi and nitrogen-fixing bacteria can complement each other and act synergistically to increase plant diversity and productivity (1, 86). Our discovery of positive correlations between *H. hongkongensis* and *Vibrio* spp. inside *H. ovalis* tissues provided a foundation for further exploration of the potentially synergistic mechanisms and physiological effects on the host seagrass.

The choice of molecular markers represents a critical consideration in microbial community profiling. While the ITS region is the most widely used locus for fungal diversity assessments (87), other fungal-specific primers targeting the 18S rRNA gene, with or without blocking oligonucleotides in PCR, could provide additional insights for certain marine fungal groups that may be less efficiently amplified by ITS-targeting primers (88–90). Our nested-PCR approach targeting the ITS2 region (via amplifications with primer pair ITS1F-ITS4, followed by fITS7-ITS4) was used to optimize fungal specificity and sensitivity while minimizing interference from host DNA co-amplification across diverse sample types. This protocol aligns with methodologies in other plant (including seagrass) mycobiome studies (18, 91) and outperformed alternative ITS-targeting primer pairs in our preliminary tests. Future comparisons of ITS and 18S rRNA gene primers in seagrass ecosystems will help to refine methodological practices for these unique environments.

### Conclusion

This is the first study to comprehensively characterize both fungal and prokaryotic communities, along with their interdomain associations, in various above- and below-ground compartments of the seagrass *H. ovalis*, from which we demonstrated the ecological importance of fungi in the seagrass ecosystem. Our results reveal that plant compartment is the most influential factor shaping the structure of the investigated microbial communities, with sampling month, pH, temperature, and salinity showing lower effects. Analyses of the diversity, taxonomic composition, network topology, and stability of fungal and prokaryotic communities collectively showed a clearly compartment-specific microbiome associated with *H. ovalis*. Future comparative studies across different locations will help to evaluate how consistently these compartment-driven patterns hold across varying geographical contexts. We hypothesize a possible symbiotic relationship between the *H. hongkongensis* (Lulworthiaceae) mycobiont and the most widely distributed Hong Kong seagrass species, enhancing our understanding of plant-fungi relationships in the marine environment. However, further studies are required to validate this hypothesis, such as co-culturing experiments, transcriptomic and genomic analyses, and fungal metabolite profiling via liquid chromatography-mass spectrometry. We also emphasize the importance of fungi in the *H. ovalis*-associated microbial networks. The findings from this study advance our knowledge of the potential roles of fungi in the seagrass ecosystem, providing foundations and hypotheses for further targeted functional verification of seagrass fungi.

## Data Availability

The fungal and prokaryotic DNA sequences amplified during this study are available in the NCBI Sequence Read Archive under BioProject number PRJNA1062533. The *Halophila ovalis* DNA sequence is available in GenBank under accession number PV202848. The fungal and prokaryotic OTU count and taxonomic information tables used in this study are available on GitHub at https://github.com/wangxiaobiol/seagrassmicrobiome.

## SUPPLEMENTAL MATERIAL

Supplemental figures aem.00606-25-s0001.pdf Figures S1 to S4.

Table S1 aem.00606-25-s0002.pdf PERMANOVA showing the effects of parameters on fungal and prokaryotic communities.

Table S2 aem.00606-25-s0003.xlsx Proportion of each OTU belonging to the family Vibrionaceae.

Table S3 aem.00606-25-s0004.xlsx Proportion of each OTU belonging to the genus *Vibrio*.

Table S4 aem.00606-25-s0005.xlsx Significantly enriched fungal taxa between compartments.

Table S5 aem.00606-25-s0006.xlsx Significantly enriched prokaryotic taxa between compartments.

Table S6 aem.00606-25-s0007.xlsx *Z*_*i*_ and *P*_*i*_ of OTUs in interdomain networks.

Table S7 aem.00606-25-s0008.xlsx Correlation and *P*-values between the key fungus *Halophilomyces hongkongensis* and prokaryotic OTUs.

ASM does not own the copyrights to Supplemental Material that may be linked to, or accessed through, an article. The authors have granted ASM a non-exclusive, world-wide license to publish the Supplemental Material files. Please contact the corresponding author directly for reuse.
