# Supplementary material for: Compartment-specific microbial communities highlight the ecological roles of fungi in a subtropical seagrass ecosystem
Source: Appl Environ Microbiol. 2025 Jul 2;91(7):e00606-25. doi: 10.1128/aem.00606-25 (PMC12285264; doi:10.1128/aem.00606-25)
Supplement: Supplemental figures — Figures S1 to S4. [file aem.00606-25-s0001.pdf]

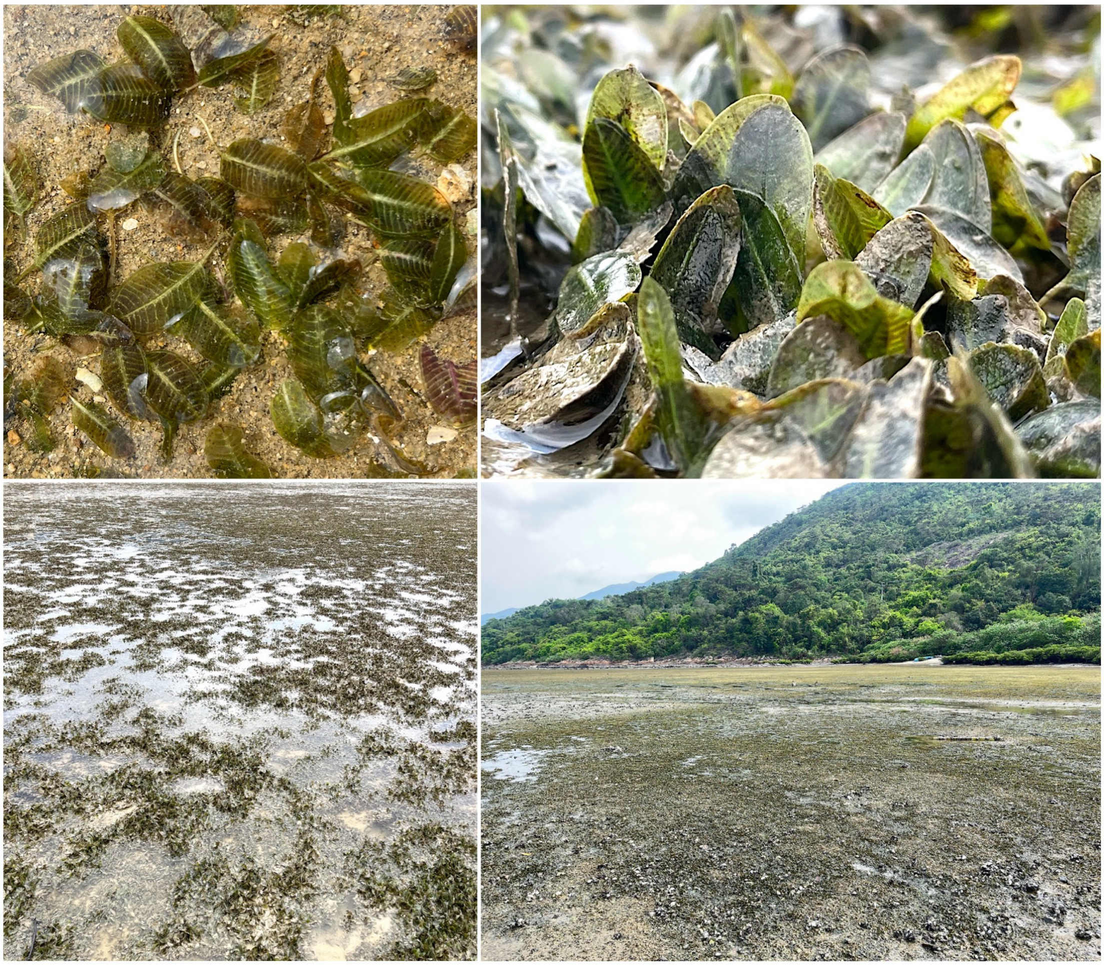

Fig. S1 High-resolution images of the *Halophila ovalis* seagrass located at an intertidal zone near San Tau Pier, in Tung Chung Bay, Lantau Island of Hong Kong.

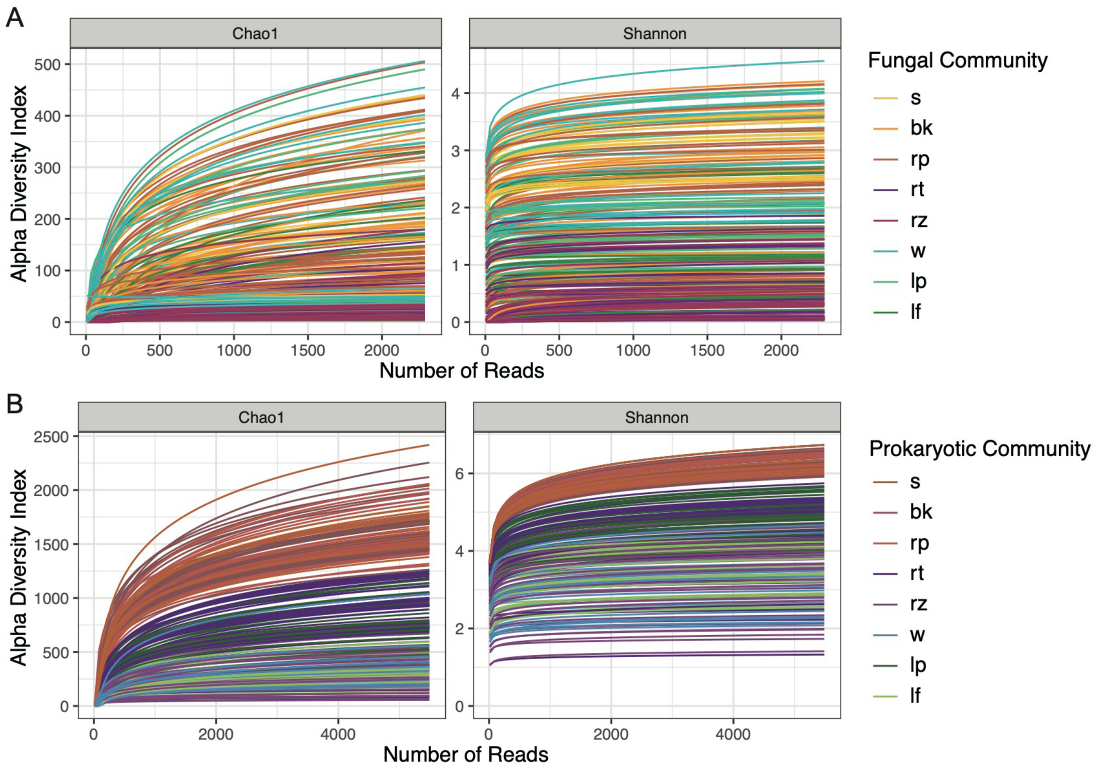

Fig. S2 Rarefaction curves of fungal (**A**) and prokaryotic (**B**) OTUs from eight compartments. Alpha-diversity indices Shannon and Chao1 were calculated for the rarefaction curves. In the compartment names, s = non-seagrass sediment, bk = bulk sediment, rp = rhizosphere sediment, rt = root, rz = rhizome, w = water, lp = leaf phylloplane, lf = leaf.

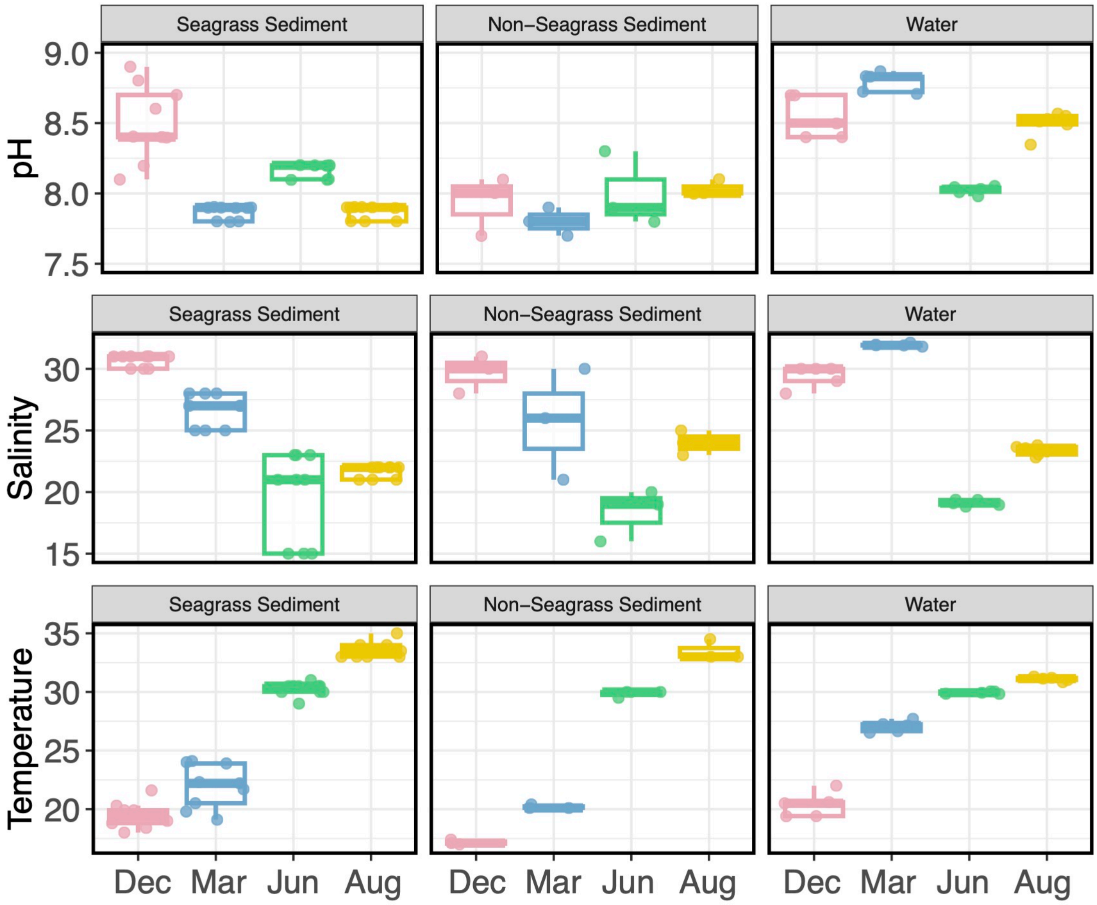

Fig. S3 Boxplots show the pH, salinity, and temperature of seagrass sediment porewater, non-seagrass sediment porewater, and seawater from four-month sampling.

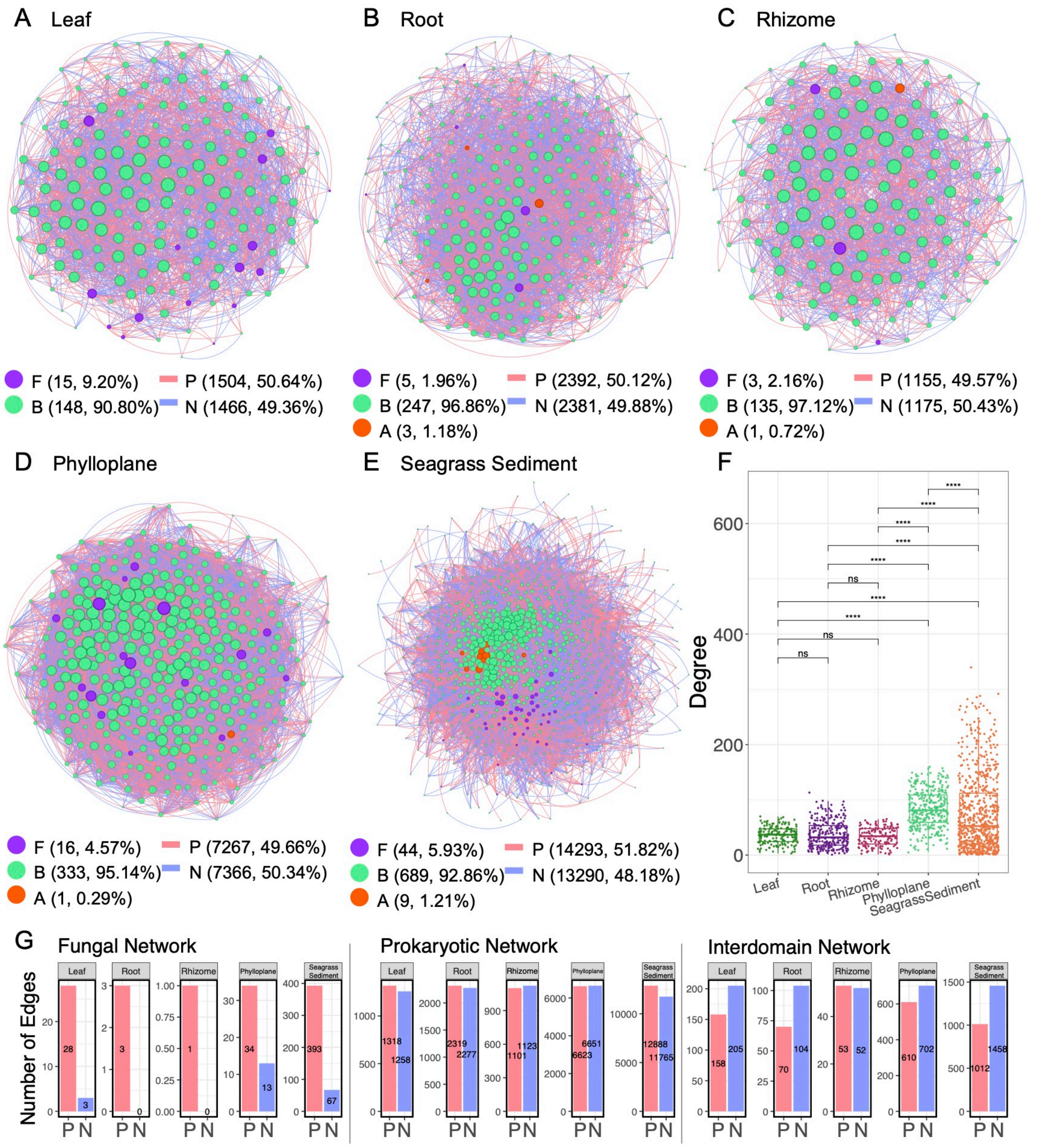

Fig. S4 Fungal-prokaryotic whole networks and properties of multiple seagrass *H. ovalis* compartments. Whole networks include all types of edges between fungi-fungi, prokaryotes-prokaryotes, and fungi-prokaryotes. (**A**–**E**) Structure of whole networks, with the number of fungal, bacterial, and archaeal nodes, as well as positive and negative edges showing in each subfigure. (**F**) Comparisons of nodes degree of the whole networks. Differences between groups evaluated by the Wilcoxon rank-sum test are indicated as: **** $P < 0.0001$, ns = not significant. (**G**) Bar plots show the number

of positive and negative edges between fungi-fungi (fungal network), prokaryotes-prokaryotes (prokaryotic network), and fungi-prokaryotes (interdomain network). In the abbreviations, F = fungi, B = bacteria, A = archaea, P = positive edges, N = negative edges.
