# Supplementary material for: Compartment-specific microbial communities highlight the ecological roles of fungi in a subtropical seagrass ecosystem
Source: Appl Environ Microbiol. 2025 Jul 2;91(7):e00606-25. doi: 10.1128/aem.00606-25 (PMC12285264; doi:10.1128/aem.00606-25)
Supplement: Table S1 — PERMANOVA showing the effects of parameters on fungal and prokaryotic communities. [file aem.00606-25-s0002.pdf]

Table S1 Permutational multivariate analysis of variance (PERMANOVA) showing the effects of the compartment, sampling month, pH, salinity, and temperature on fungal and prokaryotic communities

Factor	Fungal Community		Prokaryotic Community	
	r^2	P	r^2	P
Compartment	0.2419	0.001	0.4868	0.001
Month	0.0747	0.001	0.0385	0.001
pH	0.0117	0.004	0.0524	0.001
Salinity	0.0190	0.001	0.0209	0.001
Temperature	0.0532	0.001	0.0105	0.001
